# SCORE and REGICOR function charts underestimate the cardiovascular risk in Spanish patients with rheumatoid arthritis

**DOI:** 10.1186/ar4271

**Published:** 2013-08-21

**Authors:** Carmen Gómez-Vaquero, Alfonso Corrales, Andrea Zacarías, Javier Rueda-Gotor, Ricardo Blanco, Carlos González-Juanatey, Javier Llorca, Miguel A González-Gay

**Affiliations:** 1 Rheumatology Division, Hospital Universitari de Bellvitge-IDIBELL, L'Hopitalet, Barcelona, Spain; 2 Rheumatology Division, Hospital Universitario Marqués de Valdecilla, IFIMAV, Avenida de Valdecilla, s/n, E-39008, Santander 39008, Spain; 3 Cardiology Division of, Hospital Lucus Augusti, Lugo, Spain; 4 Division of Epidemiology and Computational Biology, School of Medicine, University of Cantabria, Santander, Spain; 5 CIBER Epidemiología y Salud Pública (CIBERESP), Santander, Spain

## Abstract

**Introduction:**

Our objective was to determine which one of the two function charts available in Spain to calculate cardiovascular (CV) risk, Systematic COronary Risk Evaluation (SCORE) or Framingham-REgistre GIroní del COR (REGICOR), should be used in patients with rheumatoid arthritis (RA).

**Methods:**

A series of RA patients seen over a one-year period without history of CV events were assessed. SCORE, REGICOR, modified (m)SCORE and mREGICOR according to the European League Against Rheumatism (EULAR) recommendations were applied. Carotid ultrasonography (US) was performed. Carotid intima-media thickness (cIMT) > 0.90 mm and/or carotid plaques were used as the gold standard test for severe subclinical atherosclerosis and high CV risk (US+). The area under the receiver operating curves (AUC) for the predicted risk for mSCORE and mREGICOR were calculated according to the presence of severe carotid US findings (US+).

**Results:**

We included 370 patients (80% women; mean age 58.9 ± 13.7 years); 36% had disease duration of 10 years or more; rheumatoid factor (RF) and/or anticyclic citrullinated peptide (anti-CCP) were positive in 68%; and 17% had extra-articular manifestations. The EULAR multiplier factor was used in 122 (33%) of the patients. The mSCORE was 2.16 ± 2.49% and the mREGICOR 4.36 ± 3.46%. Regarding US results, 196 (53%) patients were US+. The AUC mSCORE was 0.798 (CI 95%: 0.752 to 0.844) and AUC mREGICOR 0.741 (95% CI; 0.691 to 0.792). However, mSCORE and mREGICOR failed to identify 88% and 91% of US+ patients. More than 50% of patients with mSCORE ≥1% or mREGICOR >1% were US+.

**Conclusions:**

Neither of these two function charts was useful in estimating CV risk in Spanish RA patients.

## Introduction

Rheumatoid arthritis (RA) is a condition associated with high risk for cardiovascular (CV) morbidity and mortality [1] due to a process of accelerated atherosclerosis [2]. It is the result of a compound effect mediated by the influence of genetic and classic CV risk factors along with inflammation [3,4]. In this regard, a chronic proinflammatory state is of pivotal importance to accentuate the role of classic CV risk factors [5]. Strong evidence reveals that chronic inflammatory markers are independently associated with CV mortality and morbidity in RA [6-9].

CV disease, especially ischemic heart disease, is one of the most frequent causes of death in Spain [10]. Nevertheless, the incidence of CV disease in general and of ischemic coronary heart disease in particular is lower than in other countries [11].

Based on a pool of datasets from 12 European cohort studies, mainly carried out in general population settings, European experts performed the Systematic COronary Risk Evaluation (SCORE) project to develop a risk scoring system for use in the clinical management of CV risk in European clinical practice [12]. The SCORE system estimates the 10-year risk of a first fatal atherosclerotic event, including heart attack, stroke or other occlusive arterial disease and sudden cardiac death. Risk estimates have been produced as charts for high and low risk regions in Europe [12]. Spain was included in the low risk group. In the Spanish population, like in other European countries, a high CV risk has been defined by a SCORE of ≥ 5%.

Framingham algorithms have also proved to be extremely useful tools for screening patients and in primary prevention in countries with a high incidence of coronary and CV disease [13]. However, they were found to overestimate the risk of coronary heart disease in some countries [14]. Therefore, a validated methodology to adapt the Framingham algorithm to each specific country was developed [15]. Currently, in Spain, apart from the SCORE function, there is another function to estimate the presence of high CV risk: the Framingham-REgistre GIroní del COR (REGICOR) calibrated function [16]. The VERIFICA study (Validation of the Adapted Framingham Individual Coronary Incident Risk Algorithm) demonstrated the reliability and precision of the REGICOR adaptation in Spain [17]. A high CV risk has been defined by a REGICOR of ≥ 10%.

Adequate stratification of the CV risk is an issue of major importance in patients with RA. A task force of the European League Against Rheumatism (EULAR) has recommended a CV risk assessment using National Guidelines for all RA patients on an annual basis [18]. In the absence of National Guidelines, the SCORE function model is recommended. Additionally, to address the excess risk of patients presenting with RA, the experts of the task force proposed to adapt the CV risk by the application of a multiplier factor of 1.5 in those patients with two of the following three criteria: disease duration of >10 years, rheumatoid factor (RF) or anticyclic citrullinated peptide (anti-CCP) antibody positivity, and the presence of certain extra-articular manifestations.

Several validated non-invasive imaging techniques are currently available to determine subclinical atherosclerosis. They can offer a unique opportunity to study the relation of surrogate markers to the development of atherosclerosis. Among them, by the assessment of carotid intima-media thickness (cIMT) and the presence of plaques, carotid ultrasonography (US) has become an affordable efficient technique to measure the presence of subclinical atherosclerosis in RA [19,20].

In the general population, cIMT was independently associated with future risk of ischemic coronary events and stroke in middle-aged and older individuals. Furthermore, the finding of an atherosclerotic plaque increased the predicted coronary artery disease risk at any level of cIMT [21-24].

Increased cIMT and increased frequency of plaques have been found in patients with RA, even in selected patients without classic CV risk factors [25,26]. In keeping with the results observed in the general population, cIMT was found to predict the development of CV events in RA. In this regard, RA patients without classic CV risk factors at the time of the US assessment who had cIMT values greater than 0.90 mm had increased risk of suffering CV events over a five-year follow-up period [27]. Evans *et al. *also confirmed the implication of carotid plaques as predictors of future CV events in RA. They found a 2.5 increased risk of CV complications among RA patients with unilateral plaques, and 4.3 among those with bilateral plaques [28]. These results highlight the potential role of carotid US as a predictor of high CV risk in RA.

Taking these considerations together, we designed a study to establish which one of the two function charts available in Spain to calculate the CV risk, SCORE or REGICOR, should be used in patients with RA, taking as the gold standard test the result of carotid US.

## Materials and methods

### Patients and study protocol

A set of 370 consecutive Spanish patients with a diagnosis of RA recruited from Hospital Universitario Marqués de Valdecilla (Santander, Spain), who were assessed for carotid US over a one-year period, were included in the present study [29]. All the patients fulfilled the 1987 American College of Rheumatology classification criteria for RA and also fulfilled the 2010 classification criteria for RA [30,31]. Patients with a history of CV events (ischemic heart disease, cerebrovascular accident, peripheral arterial disease or heart failure) were excluded from the study. Then, clinical records of all patients were again reviewed in an attempt to fully establish comorbidities.

A subject's written consent was obtained in all the cases. The study was approved by the Ethical Committee of the Hospital Universitario Marqués de Valdecilla (Ethical Committee of Cantabria, Spain).

### CV risk assessment

The SCORE CV risk index was calculated according to data on the age at the time of the study, sex, smoking history, the systolic arterial blood pressure and atherogenic index (total serum cholesterol level/high density lipoprotein serum (HDL) cholesterol). In parallel, the REGICOR CV risk index was calculated with age, sex, total cholesterol and HDL-cholesterol, systolic and diastolic arterial blood pressure, and history of diabetes mellitus and smoking.

Both modified (m)SCORE and mREGICOR were calculated according to the EULAR recommendations, as described above [18].

### Carotid US examination

Carotid US examination included the measurement of cIMT in the common carotid artery and the detection of focal plaques in the extracranial carotid tree. A commercially available scanner, Mylab 70, Esaote (Genoa, Italy) equipped with a 7 to 12 MHz linear transducer and the automated software guided technique radiofrequency - Quality Intima Media Thickness in real-time (QIMT, Esaote, Maastricht, The Netherlands) - was used [29]. The plaque criteria in the accessible extracranial carotid tree (common carotid artery, bulb and internal carotid artery) were focal protrusion in the lumen at least cIMT > 1.5 mm, protrusion at least 50% greater than the surrounding cIMT, or arterial lumen encroaching > 0.5 mm, according to the Mannheim consensus criteria [32]. Carotid plaques were counted in each territory and defined as no plaque, unilateral plaque or bilateral plaques. We considered cIMT > 0.90 mm and/or carotid plaques as the gold standard for severe subclinical atherosclerosis [29]. For the purpose of the present study, patients with any of these severe US findings were defined as US+.

### Statistical analysis

Results were expressed as number (percentage) or mean ± standard deviation (SD).

The area under the receiver operating curve (AUC) for the predicted risk both by mSCORE and mREGICOR was calculated according to the presence of severe carotid US findings (US+).

Using the same gold standard, we also calculated the sensitivity, specificity, positive predictive value and negative predictive value for each integer between 1% and 5% for mSCORE and between 1% and 10% for mREGICOR.

All statistical tests were performed with the package Stata V.12/SE (Stata Corp, College Station, TX, USA).

## Results

Most patients from this series of 370 patients without CV events were women (298; 80%). The age at the time of the study and disease duration was 58.9 ± 13.7 and 9.8 ± 8.3 years, respectively. One hundred thirty-four patients (36%) had a disease duration of ≥10 years; RF and/or anti-CCP positivity were found in 250 (68%) cases; a total of 61 subjects (17%) had extra-articular manifestations (nodular disease in 24 patients, secondary Sjögren's syndrome in 21, pleuritis/pericarditis in 8, pulmonary fibrosis in 7, Raynaud's phenomenon in 3, rheumatoid vasculitis in 2, scleritis/episcleritis in 2, sclerosing cholangitis in 1 case and Felty's syndrome in 1 case).

The EULAR multiplier factor was required to be used in 122 (33%) of the patients. Due to this, the mSCORE was 2.16 ± 2.49% and the mREGICOR, 4.36 ± 3.46%. Regarding US results, cIMT >0.90 mm was observed in 46 (12%) patients and carotid plaques in 195 (53%). Interestingly, 45 of the 46 patients with cIMT >0.90 also had carotid plaques. According to our previous definition, 196 (53%) patients were US+.

**Figure 1:**
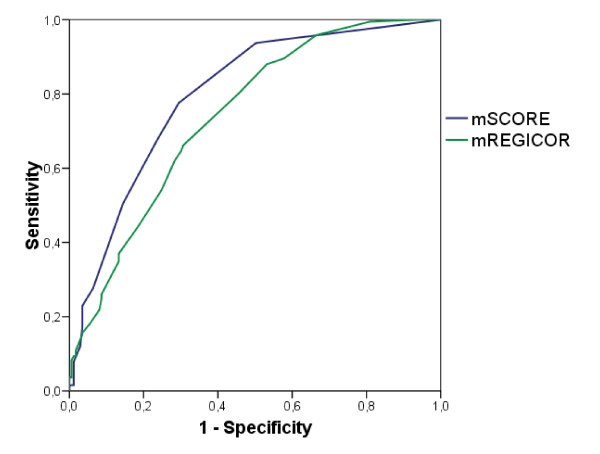
**Area under the receiver operating curves for the predicted CV risk by mSCORE and mREGICOR according to the established gold standard (US+)**. CV, cardiovascular; mREGICOR, modified Framingham-REgistre GIroní del COR; mSCORE, modified Systematic COronary Risk Evaluation; US, ultrasound

Although the values of the area under the receiver operating curves of both functions in their modified versions - AUC mSCORE: 0.798 (CI 95%: 0.752 to 0.844) and AUC mREGICOR: 0.741 (CI 95%; 0.691 to 0.792) (Figure 1) were promising with respect to their discriminating capacity, the behavior of both functions in the identification of the patients with subclinical atherosclerosis and, therefore, high CV risk was disappointing. In this regard, the mSCORE and the mREGICOR only classified 11% and 6% as patients with high CV risk, respectively. Inversely, mSCORE and mREGICOR misclassified as having high CV risk 4% and 2% of the patients without subclinical atherosclerosis. Also, the mSCORE and the mREGICOR failed to classify as having high CV risk 82% and 91% of the patients, respectively. Therefore, neither of these two functions to estimate CV risk was proved to be effective in Spanish RA patients.

More than 50% of patients with an mSCORE ≥ 1% or mREGICOR > 1% had US+. In consequence, to disclose the presence of severe subclinical atherosclerotic disease in at least 50% of these patients, carotid US would have to be performed on 73% and 86% of them, respectively.

In Table 1, we describe the sensitivity, specificity, positive predictive value and negative predictive value for each integer between 1% and 5% for mSCORE and between 1% and 10% for mREGICOR.

**Table 1:** Sensitivity, specificity, positive predictive value and negative predictive value for each integer between 1% and 5% for mSCORE and between 1% and 10% for mREGICOR

Threshold		Sensibility	Specificity	Positive predictive value	Negative predictive value
mSCORE	≥1% (n: 271)	0.939	0.500	0.679	0.879
	
	≥2% (n: 174)	0.679	0.764	0.764	0.679
	
	≥3% (n: 125)	0.510	0.856	0.800	0.608
	
	≥4% (n: 66)	0.281	0.937	0.833	0.536
	
	≥5% (n: 45)	0.179	0,966	0.854	0.511

mREGICOR	≥1% (n: 355)	-	0.058	0.541	-
	
	≥2% (n: 299)	0.958	0.335	0.615	0.879
	
	≥3% (n: 233)	0.802	0.543	0.661	0.712
	
	≥4% (n: 168)	0.620	0.717	0.708	0.629
	
	≥5% (n: 117)	0.443	0.815	0.726	0.569
	
	≥6% (n: 90)	0.349	0.867	0.744	0.545
	
	≥7% (n: 63)	0.250	0.913	0.762	0.523
	
	≥8% (n: 43)	0.177	0.948	0.791	0.509
	
	≥9% (n: 35)	0.151	0.965	0.829	0.506
	
	≥10% (n: 21)	0.094	0.983	0.857	0.495

mREGICOR, modified Framingham-REgistre GIroní del COR; mSCORE, modified Systematic COronary Risk Evaluation

Since many rheumatologists do not have access to carotid US in their daily clinical practice, we tried to find the CV risk threshold that may be useful in indicating high risk and, therefore, the need of a lipid lowering or an antihypertensive treatment.

Patients with type 1 diabetes with target organ damage, type 2 diabetes mellitus or those who have severe chronic kidney disease are considered as having very high CV risk according to current guidelines, regardless yof chart function results [24]. Forty three patients from this series had type 2 diabetes or chronic renal disease and were excluded from further analyses. Interestingly, 34 (79%) of them also had severe US findings, that is, US+.

When we used lower thresholds of mSCORE and mREGICOR than those usually recommended in the 327 RA patients without diabetes or severe chronic kidney disease, we increased the number of patients classified as having high CV. Using a threshold of 3% for mSCORE or 4% for mREGICOR, we identified more than 50% of US+ patients, even at the risk of misclassification as high CV risk in 20 to 30% of the patients without severe subclinical atherosclerosis. Specifically, an mSCORE ≥ 3% classified correctly as having high CV risk in 55% of patients and incorrectly in 18% of patients. Likewise, an mREGICOR ≥ 4% classified as having high CV correctly in 63% of patients and incorrectly in 30% of patients.

## Discussion

Comparisons between SCORE and REGICOR did not disclose that one of them might be better than the other in establishing the CV risk in Spanish individuals without rheumatic diseases [33-37]. In the present study, neither mSCORE nor mREGICOR were shown to be effective in the stratification of the CV risk of Spanish RA patients. In this regard, when CV risk was defined by the presence of carotid subclinical atherosclerosis, we observed that both CV risk function charts identified a very low proportion of RA patients with high CV risk and misclassified as having high CV risk a substantial proportion of the patients without subclinical atherosclerosis. Nevertheless, SCORE and REGICOR predict CV mortality and CV events, respectively, and none of them has been tested for the prediction of subclinical atherosclerosis. In addition, these two CV risk function charts have been designed to detect more than just atherosclerotic disease. Therefore, some of the apparently misclassified patients might not truly be misclassified if other CV outcomes were considered in this study.

This study has some limitations. First, actual CV events and mortality were not available and carotid US was used as a surrogate marker of CV risk. Second, our results cannot be generalized to other populations within and outside Spain.

Several years ago, del Rincon *et al. *stated that physicians who provide care to individuals with RA should be aware of the increased risk of CV events in these patients and implement appropriate diagnostic and therapeutic measures [38]. More recently, Crowson and Gabriel emphasized that the CV risk assessment tools designed for the general population may not accurately estimate the CV risk for individual patients with RA, even if the multiplication factor indicated by the EULAR task force is applied [39]. These authors concluded that although in some circumstances patients with RA satisfying the criteria for use of the multiplier factors may have increased risk of CV disease, some others that do not meet these criteria may also have increased CV risk [39].

A good method of predicting CV events would allow us to establish a high risk threshold to identify, with high sensitivity and specificity, those patients who have a high probability of presenting a CV event; and also a low risk threshold below which we could establish a very low probability of having a CV event. With the help of these two thresholds, we would have to set up a strategy consisting of an indication of therapy to patients who are above the threshold of high CV risk, no specific therapy to those patients who are below the low risk threshold, and the need for performing a diagnostic test in those patients included in the category of moderate/intermediate risk.

Recent studies indicate that carotid US may be a useful diagnostic tool to disclose subclinical atherosclerosis [29], but universal screening is not always feasible in many rheumatology clinics. Therefore, it would be interesting to define a CV risk threshold above which the indication of intensive lipid lowering or antihypertensive treatment was required in patients with RA.

Patients with type 1 diabetes with target organ damage, type 2 diabetes mellitus or those with severe chronic kidney disease (if glomerular filtration rate <60 ml/min/1.73 m2) are considered as having very high CV risk according to current guidelines [24]. In keeping with that, 79% of the RA patients with diabetes or severe kidney disease of our study were US+. Therefore, these patients are considered as having high CV risk and, because of that, non-invasive techniques used to detect the presence of subclinical atherosclerosis would not be required in these patients. According to our present results, in the remaining patients, SCORE and REGICOR do not seem to be useful in identifying RA Spanish patients with subclinical atherosclerosis and increased CV risk.

## Conclusions

Neither of the two function charts used in Spain to estimate the CV risk in the general population proved to be effective in stratifying the CV risk in patients with RA. Nevertheless, further assessment of the accuracy of the SCORE to identify high CV risk RA patients is needed in other populations.

## Abbreviations

anti-CCP: anticyclic citrullinated peptide; AUC: area under the receiver operating curve; cIMT: carotid intima-media thickness; CV: cardiovascular; EULAR: European League Against Rheumatism; HDL: high density lipoprotein serum; m: modified; RA: rheumatoid arthritis; REGICOR: Framingham-REgistre GIroní del COR; RF: rheumatoid factor; SCORE: Systematic COronary Risk Evaluation; SD: standard deviation; US: ultrasonography; VERIFICA: Validation of the adapted Framingham individual coronary incident risk algorithm.

## Competing interests

The authors declare that they have no competing interests.

## Authors' contributions

CGV made substantial contributions to the conception and design of the study, data analysis, and substantial contributions to the elaboration of the manuscript. AC performed the US study, contributed to the elaboration of the protocol of study, and helped in the interpretation of data. AZ performed data analysis and helped to draft the manuscript. JR and RB recruited patients for the study. CGJ contributed to the elaboration of the protocol of study, and helped in the interpretation of data. AC, JR, RB and CGJ took part in the elaboration of the manuscript. JL participated in the design of the study, data analysis and helped to draft the manuscript. MAGG has made substantial contributions to conception and design of the study, acquisition of data, coordination of the study, and helped to draft the manuscript and was responsible of the final drafting and elaboration of the manuscript. All authors read and approved the final version of the manuscript for publication. CGV, JL and MAGG share senior authorship of this manuscript.

## References

[B1] Aviña-Zubieta JA Choi HK Sadatsafavi M Etminan M Esdaile JM Lacaille D Risk of CV mortality in patients with rheumatoid arthritis: a meta-analysis of observational studies Arthritis Rheum 2008 15 1690 1697 10.1002/art.24092 19035419

[B2] Gonzalez-Gay MA Gonzalez-Juanatey C Martin J Rheumatoid arthritis: a disease associated with accelerated atherogenesis Semin Arthritis Rheum 2005 15 8 17 10.1016/j.semarthrit.2005.03.004 16084219

[B3] Rodríguez-Rodríguez L González-Juanatey C García-Bermúdez M Vázquez-Rodríguez TR Miranda-Filloy JA Fernández-Gutiérrez B Llorca J Martin J González-Gay MA CCR5Δ32 variant and cardiovascular disease in patients with rheumatoid arthritis: a cohort study Arthritis Res Ther 2011 15 R133 10.1186/ar3444 21846359 PMC3239375

[B4] Dessein PH Joffe BI Veller MG Stevens BA Tobias M Reddi K Stanwix AE Traditional and nontraditional cardiovascular risk factors are associated with atherosclerosis in rheumatoid arthritis J Rheumatol 2005 15 435 442 15742434

[B5] Sattar N McCarey DW Capell H McInnes IB Explaining how "high-grade" msystemic inflammation accelerates vascular risk in rheumatoid arthritis Circulation 2003 15 2957 2963 10.1161/01.CIR.0000099844.31524.05 14676136

[B6] Gonzalez-Gay MA Gonzalez-Juanatey C Lopez-Diaz MJ Piñeiro A Garcia-Porrua C Miranda-Filloy JA Ollier WE Martin J Llorca J HLA-DRB1 and persistent chronic inflammation contribute to CV events and CV mortality in patients with rheumatoid arthritis Arthritis Rheum 2007 15 125 132 10.1002/art.22482 17266100

[B7] Goodson NJ Symmons DP Scott DG Bunn D Lunt M Silman AJ Baseline levels of C-reactive protein and prediction of death from CV disease in patients with inflammatory polyarthritis: a ten-year followup study of a primary care-based inception cohort Arthritis Rheum 2005 15 2293 2299 10.1002/art.21204 16052597

[B8] Jacobsson LT Turesson C Hanson RL Pillemer S Sievers ML Pettitt DJ Bennett PH Knowler WC Joint swelling as a predictor of death from CV disease in a population study of Pima Indians Arthritis Rheum 2001 15 1170 1176 10.1002/1529-0131(200105)44:5<1170::AID-ANR200>3.0.CO;2-T 11352251

[B9] Wållberg-Jonsson S Johansson H Ohman ML Rantapää-Dahlqvist S Extent of inflammation predicts CV disease and overall mortality in seropositive rheumatoid arthritis. A retrospective cohort study from disease onset J Rheumatol 1999 15 2562 2571 10606363

[B10] Marrugat J Elosúa R Martí H Epidemiology of ischaemic heart disease in Spain: estimation of the number of cases and trends from 1997 to 2005 Rev Esp Cardiol 2002 15 337 346 10.1016/S0300-8932(02)76611-6 11975899

[B11] Sans S Kesteloot H Kromhout D The burden of CV diseases mortality in Europe. Task force of the European Society of Cardiology on CV Mortality and Morbidity Statistics in Europe Eur Heart J 1997 15 1231 1248 10.1093/oxfordjournals.eurheartj.a015434 9458415

[B12] Conroy RM Pyörälä K Fitzgerald AP Sans S Menotti A De Backer G De Bacquer D Ducimetière P Jousilahti P Keil U Njølstad I Oganov RG Thomsen T Tunstall-Pedoe H Tverdal A Wedel H Whincup P Wilhelmsen L Graham IM SCORE project group Estimation of ten-year risk of fatal cardiovascular disease in Europe: the SCORE project Eur Heart J 2003 15 987 1003 10.1016/S0195-668X(03)00114-3 12788299

[B13] Kannel WB D'Agostino RB Sullivan L Wilson PW Concept and usefulness of CV risk profiles Am Heart J 2004 15 16 26 10.1016/j.ahj.2003.10.022 15215787

[B14] Marrugat J D'Agostino R Sullivan L Elosua R Wilson P Ordovas J Solanas P Cordón F Ramos R Sala J Masiá R Kannel WB An adaptation of the Framingham coronary heart disease risk function to European Mediterranean areas J Epidemiol Community Health 2003 15 634 638 10.1136/jech.57.8.634 12883073 PMC1732543

[B15] D'Agostino RB Grundy S Sullivan LM Wilson P The CHD Risk Prediction Group Validation of the Framingham coronary heart disease prediction scores: results of a multiple ethnic groups investigation JAMA 2001 15 180 187 10.1001/jama.286.2.180 11448281

[B16] Ramos R Solanas P Cordón F Rohlfs I Elosua R Sala J Masiá R Faixedas MT Marrugat J Comparison of population coronary heart disease risk estimated by the Framingham original and REGICOR calibrated functions Med Clin (Barc) 2003 15 521 526 14599406 10.1016/s0025-7753(03)74007-x

[B17] Marrugat J Subirana I Comín E Cabezas C Vila J Elosua R Nam BH Ramos R Sala J Solanas P Cordón F Gené-Badia J D'Agostino RB VERIFICA Investigators Validity of an adaptation of the Framingham CV risk function: the VERIFICA study J Epidemiol Community Health 2007 15 40 47 10.1136/jech.2005.038505 17183014 PMC2465597

[B18] Peters MJ Symmons DP McCarey D Dijkmans BA Nicola P Kvien TK McInnes IB Haentzschel H Gonzalez-Gay MA Provan S Semb A Sidiropoulos P Kitas G Smulders YM Soubrier M Szekanecz Z Sattar N Nurmohamed MT EULAR evidence-based recommendations for CV risk management in patients with rheumatoid arthritis and other forms of inflammatory arthritis Ann Rheum Dis 2010 15 325 331 10.1136/ard.2009.113696 19773290

[B19] Kerekes G Soltész P Nurmohamed MT Gonzalez-Gay MA Turiel M Végh E Shoenfeld Y McInnes I Szekanecz Z Validated methods for assessment of subclinical atherosclerosis in rheumatology Nat Rev Rheumatol 2012 15 224 234 10.1038/nrrheum.2012.16 22349611

[B20] Gonzalez-Gay MA Gonzalez-Juanatey C Vazquez-Rodriguez TR Martin J Llorca J Endothelial dysfunction, carotid intima-media thickness, and accelerated atherosclerosis in rheumatoid arthritis Semin Arthritis Rheum 2008 15 67 70 10.1016/j.semarthrit.2008.02.001 18395772

[B21] Belcaro G Nicolaides AN Ramaswami G Cesarone MR De Sanctis M Incandela L Ferrari P Geroulakos G Barsotti A Griffin M Dhanjil S Sabetai M Bucci M Martines G Carotid and femoral ultrasound morphology screening and CV events in low risk subjects: a 10-year follow-up study (the CAFES-CAVE study (1)) Atherosclerosis 2001 15 379 387 10.1016/S0021-9150(00)00665-1 11395035

[B22] Nambi V Chambless L Folsom AR He M Hu Y Mosley T Volcik K Boerwinkle E Ballantyne CM Carotid intima-media thickness and presence or absence of plaque improves prediction of coronary heart disease risk: the ARIC (Atherosclerosis Risk In Communities) study J Am Coll Cardiol 2010 15 1600 1607 10.1016/j.jacc.2009.11.075 20378078 PMC2862308

[B23] Mancia G De Backer G Dominiczak A Cifkova R Fagard R Germano G Grassi G Heagerty AM Kjeldsen SE Laurent S Narkiewicz K Ruilope L Rynkiewicz A Schmieder RE Boudier HA Zanchetti A Vahanian A Camm J De Caterina R Dean V Dickstein K Filippatos G Funck-Brentano C Hellemans I Kristensen SD McGregor K Sechtem U Silber S Tendera M Widimsky P 2007 Guidelines for the Management of Arterial Hypertension: the Task Force for the Management of Arterial Hypertension of the European Society of Hypertension (ESH) and of the European Society of Cardiology (ESC) J Hypertens 2007 15 1105 1187 10.1097/HJH.0b013e3281fc975a 17563527

[B24] European Association for Cardiovascular Prevention & Rehabilitation Reiner Z Catapano AL De Backer G Graham I Taskinen MR Wiklund O Agewall S Alegria E Chapman MJ Durrington P Erdine S Halcox J Hobbs R Kjekshus J Filardi PP Riccardi G Storey RF Wood D ESC Committee for Practice Guidelines (CPG) 2008-2010 and 2010-2012 Committees ESC/EAS Guidelines for the management of dyslipidaemias: the Task Force for the management of dyslipidaemias of the European Society of Cardiology (ESC) and the European Atherosclerosis Society (EAS) Eur Heart J 2011 15 1769 1818 21712404 10.1093/eurheartj/ehr158

[B25] van Sijl AM Peters MJ Knol DK de Vet HC Gonzalez-Gay MA Smulders YM Dijkmans BA Nurmohamed MT Carotid intima media thickness in rheumatoid arthritis as compared to control subjects: a meta-analysis Semin Arthritis Rheum 2011 15 389 397 10.1016/j.semarthrit.2010.06.006 20889191

[B26] Gonzalez-Juanatey C Llorca J Testa A Revuelta J Garcia-Porrua C Gonzalez-Gay MA Increased prevalence of severe subclinical atherosclerotic findings in long-term treated rheumatoid arthritis patients without clinically evident atherosclerotic disease Medicine (Baltimore) 2003 15 407 413 10.1097/01.md.0000101572.76273.60 14663290

[B27] Gonzalez-Juanatey C Llorca J Martin J Gonzalez-Gay MA Carotid intima-media thickness predicts the development of CV events in patients with rheumatoid arthritis Semin Arthritis Rheum 2009 15 366 371 10.1016/j.semarthrit.2008.01.012 18336869

[B28] Evans MR Escalante A Battafarano DF Freeman GL O'Leary DH del Rincón I Carotid atherosclerosis predicts incident acute coronary syndromes in rheumatoid arthritis Arthritis Rheum 2011 15 1211 1220 10.1002/art.30265 21305526 PMC3286362

[B29] Corrales A González-Juanatey C Peiró ME Blanco R Llorca J González-Gay MA Carotid ultrasound is useful for the cardiovascular risk stratification of patients with rheumatoid arthritis: results of a population-based study Ann Rheum Dis 2013 in press 10.1136/annrheumdis-2012-203101 23505241

[B30] Arnett FC Edworthy SM Bloch DA McShane DJ Fries JF Cooper NS Healey LA Kaplan SR Liang MH Luthra HS Medsger TA Jr Mitchell DM Neustadt DH Pinals RS Schaller JG Sharp JT Wilder RL Hunder GG The American Rheumatism Association 1987 revised criteria for the classification of rheumatoid arthritis Arthritis Rheum 1988 15 315 324 10.1002/art.1780310302 3358796

[B31] Aletaha D Neogi T Silman AJ Funovits J Felson DT Bingham CO 3rd Birnbaum NS Burmester GR Bykerk VP Cohen MD Combe B Costenbader KH Dougados M Emery P Ferraccioli G Hazes JM Hobbs K Huizinga TW Kavanaugh A Kay J Kvien TK Laing T Mease P Ménard HA Moreland LW Naden RL Pincus T Smolen JS Stanislawska-Biernat E Symmons D 2010 rheumatoid arthritis classification criteria: an American College of Rheumatology/European League Against Rheumatism collaborative initiative Ann Rheum Dis 2010 15 1580 1588 10.1136/ard.2010.138461 20699241

[B32] Touboul PJ Hennerici MG Meairs S Adams H Amarenco P Bornstein N Csiba L Desvarieux M Ebrahim S Fatar M Hernandez Hernandez R Jaff M Kownator S Prati P Rundek T Sitzer M Schminke U Tardif JC Taylor A Vicaut E Woo KS Zannad F Zureik M Mannheim carotid intima-media thickness consensus (2004-2006). An update on behalf of the Advisory Board of the 3rd and 4th Watching the Risk Symposium, 13th and 15th European Stroke Conferences, Mannheim, Germany, 2004, and Brussels, Belgium, 2006 Cerebrovasc Dis 2007 15 75 80 10.1159/000097034 17108679

[B33] Baena Díez JM del Val Garcia JL Héctor Salas Gaetgens L Sánchez Pérez R Altes Vaques E Deixens Martínez B Amatller Corominas M Katia Núñez Casillas D Comparison of the SCORE and REGICOR models for calculating CV risk in CV disease-free individuals at a healthcare center in Barcelona, Spain Rev Esp Salud Publica 2005 15 453 464 10.1590/S1135-57272005000400003 16465962

[B34] Buitrago F Cañón-Barroso L Díaz-Herrera N Cruces-Muro E Escobar-Fernández M Serrano-Arias JM Comparison of the REGICOR and SCORE function charts for classifying CV risk and for selecting patients for hypolipidemic or antihypertensive treatment Rev Esp Cardiol 2007 15 139 147 10.1157/13099460 17338879

[B35] Gil-Guillén V Orozco-Beltrán D Maiques-Galán A Aznar-Vicente J Navarro J Cea-Calvo L Quirce-Andrés F Redón J Merino-Sánchez J Agreement between REGICOR and SCORE scales in identifying high CV risk in the Spanish population Rev Esp Cardiol 2007 15 1042 1050 10.1157/13111236 17953925

[B36] Comín E Solanas P Cabezas C Subirana I Ramos R Gené-Badía J Cordón F Grau M Cabré-Vila JJ Marrugat J Estimating CV risk in Spain using different algorithms Rev Esp Cardiol 2007 15 693 702 10.1157/13108274 17663853

[B37] Buitrago Ramírez F Cañón Barroso L Díaz Herrera N Cruces Muro E Bravo Simón B Pérez Sánchez I Comparison of the SCORE function chart and the Framingham-REGICOR equation to estimate the CV risk in an urban population after 10 years of follow-up Med Clin (Barc) 2006 15 368 373 10.1157/13092437 16987481

[B38] del Rincón ID Williams K Stern MP Freeman GL Escalante A High incidence of CV events in a RA cohort not explained by traditional cardiac risk factors Arthritis Rheum 2001 15 2737 2745 10.1002/1529-0131(200112)44:12<2737::AID-ART460>3.0.CO;2-# 11762933

[B39] Crowson CS Gabriel SE Towards improving cardiovascular risk management in patients with rheumatoid arthritis: the need for accurate risk assessment Ann Rheum Dis 2011 15 719 721 10.1136/ard.2010.145482 21345812 PMC3907170

